# Editorial: Editors’ showcase: insights in cancer cell biology

**DOI:** 10.3389/fcell.2024.1459030

**Published:** 2024-07-29

**Authors:** Geoffrey Brown, Andrew Liss

**Affiliations:** ^1^ School of Biomedical Sciences, Institute of Clinical Sciences, University of Birmingham, Birmingham, United Kingdom; ^2^ Cancer Centre, Mass General Research Institute, Harvard Medical School, Boston, MA, United States; ^3^ Department of Surgery, Massachusetts General Hospital, Harvard Medical School, Boston, MA, United States

**Keywords:** cancer stem cells, leukemia, lymphoma, oncogenes, immunotherapy

Significant progress has been made regarding the treatment of many cancers, but challenges remain. One in two people will develop cancer, mostly in old age and the need is to move away from systemic cytotoxic therapies. Treatment of advanced and metastatic disease has not improved substantially for 40 years with many of these cancers viewed as untreatable. A key to unlocking new treatments for advanced cancers is the existence of cancer stem cells (CSCs). They sustain a cancer by producing a hierarchy of differentiated or poorly differentiated cells and seem to be responsible for aggressive and metastatic disease. CSCs are insensitive to chemotherapy and persist giving rise to disease relapse. Desmoplastic small round cell tumour (DSRCT), which is driven by EWSC1-WT1 fusion oncogene, is a rapidly progressing pediatric cancer that is not curative. Magrath et al. have examined the existence of CSCs within DSRCT and shown that expression of stemness markers (*SOX2* and *NANOG*) relates to a worse patient survival and elevated expression for metastatic DSRCT. They modelled CSCs *in vitro* by establishing tumour spheres that have an increased level of stemness markers. Though resistant to doxorubicin, tumour spheres were sensitive to EWSC1-WT1 knockdown leading to the possibility of targeting to eradicate DSRCT CSCs.

To what extent the behaviour of CSCs differs from that of normal stem cells is all important to killing CSCs and sparing normal stem cells. The sustained production of a hierarchy of leukaemia cells by leukaemia stem cells (LSC) is akin to generating various blood cells by haematopoietic stem cells (HSCs). In new models for haematopoiesis, HSCs choose a cell lineage directly from a spectrum of all end-cell options. Their developmental trajectories are progressive and broad whereby HSCs can still veer towards an adjacent option. Whilst many leukaemias are likely to arise from a HSC, they are categorised based on the cells belonging to a cell lineage. Chronic myeloid leukaemia (CML) arises from transformation of a HSC and, by contrast to the cell of origin, the progeny of CML LSCs is restricted largely to neutrophils. Brown examines the view that some leukaemia signature oncogenes guide and restrict the cell lineage upon transformation of a HSC/progenitor cell.

Tongue squamous cell carcinoma is an aggressive and highly metastatic cancer. To find druggable targets for patients who are resistant to neoadjuvant therapy, George et al. compared the proteomic and phospho-proteomic profiles for primary tumors from resistant and sensitive patients. These analyses revealed a signature for neoadjuvant resistance. MAPK1, AKT1, and MAPK3, enrichment of Rho GTPase signalling and hyperphosphorylation of proteins that are involved in cell mobility, invasion, and drug resistance were predictive of resistance. Differences in the phosphorylation of keratins (KRT10 and KRT1) were also observed.

The cytokine VEGF-A is the most potent stimulant of angiogenesis and is a major therapeutic target for cancer because angiogenesis is important to tumour growth. The predominant isoform of VEGF-A is VEGF165, and its heparin-binding domain (HBD) plays a critical role in mitogenicity. Takada et al. have shown that integrin αvβ3 binds to the isolated heparin-binding domain of VEGF165 and discovered that VEGF165 binds to VEGFR2/KDR domain 1 (D1, in addition to domains 2 and 3). They identified the VEGF165 HBD amino acids that are required for these interactions. A VEGF165 mutant that was defective in αvβ3 and KDR D1 was unable to induce the phosphorylation of ERK1/2 and integrin β3 or support endothelial proliferation. From other findings, Takada et al. have argued that integrin αvβ3 is a negative regulator of VEGF165 signalling which is supported by integrin αvβ3 knockout mice having VEGF165 enhanced signalling.

Re et al. summarise advances regarding promising news drugs for the treatment of pediatric and adolescent patients with Hodgkin lymphoma. Current strategies have achieved a better balance between supporting survival versus the long-term toxicities. Even so, Re et al. concluded that there is a need to look at risk classification based on the additional use of PET imaging, identify patients for proton therapy, and for new drugs from the clarification of appropriate targets.

Telomerase activity is often low/undetectable in normal cells. Telomerase reactivation occurs frequently in several cancers, leading to telomere elongation which has long been seen to facilitate unlimited growth of cancer cells. Point mutations in the core promotor region of the *telomerase reverse transcriptase* (*TERT)* gene, which promote gene expression, are considered to cause telomerase reactivation within cancer cells and Tornesello et al. review their roles in cancer. They conclude that strategies that specifically target *TERT* gene promoter mutations are likely to have an impact on cancers that bear such mutations. Small molecules have been identified that are able to reestablish silencing of the *TERT* gene.

The success of COVID-19 mRNA vaccines has led to a renewed interest in immunotherapy for cancer regarding vaccines that are specific for a patient’s cancer to prevent disease recurrence. Regarding vaccines, T helper 2 (CD4^+^) cells play a complex role in the progression of breast cancer and can drive terminal differentiation to block carcinogenesis. The paper by Boieri et al. has shown that thymic stromal lymphopoietin-stimulated CD4^+^ T cells exert an anticancer effect in advanced breast cancer. The stimulated cells drove breast cancer cells into senescence via the release of interferon γ and tumour necrosis factor α, which bound directly to the receptors on the breast cancer cells. This novel mechanism adds to the armoury of cancer immunotherapy.

From the above, there is still a need to search for new anticancer strategies based on exploiting areas that have been known for decades such as targeting intracellular signalling pathways, telomerase, and angiogenesis, as well as activation of the immune response. The most pressing matter is to find means to kill CSCs which requires exploring the physiology of normal stem cells versus CSCs in more detail to provide novel therapeutic avenues.

